# Short-Term Hypoxia in Cells Induces Expression of Genes Which Are Enhanced in Stressed Cells

**DOI:** 10.3390/genes13091596

**Published:** 2022-09-06

**Authors:** Inga Peciuliene, Egle Jakubauskiene, Laurynas Vilys, Ruta Zinkeviciute, Kotryna Kvedaraviciute, Arvydas Kanopka

**Affiliations:** 1 Department of Immunology and Cell Biology, Institute of Biotechnology, Life Sciences Center, Vilnius University, LT 10257 Vilnius, Lithuania; 2 Department of Eukaryote Gene Engineering, Institute of Biotechnology, Life Sciences Center, Vilnius University, LT 10257 Vilnius, Lithuania; 3 Department of Biological DNA Modification, Institute of Biotechnology, Life Sciences Center, Vilnius University, LT 10257 Vilnius, Lithuania

**Keywords:** hypoxia, splicing, RNA, kinases, phosphatases, SR proteins

## Abstract

All living organisms must respond to, and defend against, environmental stresses. Depending on the extent and severity of stress, cells try to alter their metabolism and adapt to a new state. Changes in alternative splicing of pre-mRNA are a crucial regulation mechanism through which cells are able to respond to a decrease in oxygen tension in the cellular environment. Currently, only limited data are available in the literature on how short-term hypoxia influences mRNA isoform formation. In this work, we discovered that expressions of the same genes that are activated during cellular stress are also activated in cells under short-term hypoxic conditions. Our results demonstrate that short-term hypoxia influences the splicing of genes associated with cell stress and apoptosis; however, the mRNA isoform formation patterns from the same pre-mRNAs in cells under short-term hypoxic conditions and prolonged hypoxia are different. Obtained data also show that short-term cellular hypoxia increases protein phosphatase but not protein kinase expression. Enhanced levels of protein phosphatase expression in cells are clearly important for changing mRNA isoform formation.

## 1. Introduction

One of the reasons for the evolutionary success of mammals is their extraordinary capacity to adapt to changing environmental conditions. All living organisms need to sense and respond to conditions that stress their homeostatic mechanisms. Many environmental variations, such as temperature shock, hypoxia, oxidative stress, nutrient deprivation, or DNA damage, impose stresses that can damage or kill the organism in the absence of an appropriate response. To minimize the effects of stress-related damage, organisms have evolved systems to detect and respond to stress [1,2]. Different stress conditions can lead to changes in gene expression patterns caused by a general shutdown and reprogramming of protein synthesis [2]. Therefore, depending on the level and mode of stress, different defense mechanisms and pro-survival strategies are mounted; however, if these are unsuccessful, then the cell death programs are activated to eliminate these damaged cells from the organism [3].

Different types of cells differently adapt to hypoxia by modulating gene expression through the stabilization of hypoxia-inducible transcription factor HIF-1 [4]. Changes in alternative splicing of pre-mRNA are another regulation mechanism through which cells are able to respond to hypoxic conditions. Along with the reprogramming of alternative splicing patterns of genes, alterations in splicing factor expression and post-translational modification have been observed under certain pathological conditions, including hypoxia. The preferable expression of certain splice variants over others is one of the major strategies co-opted by cells while adapting to hypoxic stress [5].

In the past few decades, substantial progress has been made in understanding the principles of cellular signal transduction. Thanks to profound genetic and proteomic analyses, the development of novel analytical techniques, and increasing ability to pharmacologically modulate cellular signal transduction, a significant number of signaling pathways have been identified, most of which have been shown to be highly conserved throughout phylogeny. In addition, most of these signaling pathways have been shown to be highly complex, both in their nature and in their regulation pattern [6].

In current work, we show that cellular processes induced in transient hypoxia are slightly different that those observed in prolonged hypoxic conditions. Our results demonstrate that during short-term hypoxic conditions, cells activate expression of the same genes that are activated in cell stress response. Moreover, our studies revealed that the mRNA isoform formation ratios from the same pre-mRNAs in cells under short-term hypoxic conditions and prolonged hypoxia are different.

## 2. Materials and Methods

*Computation analysis from public sequencing database.* Raw sequencing data of HCT116 cells were downloaded from NCBI SRA (Bioproject ID PRJNA321835, [7]. Raw read quality was evaluated using FASTQC (https://www.bioinformatics.babraham.ac.uk/projects/fastqc/ (accessed on 1 March 2020), and reads were filtered by BBDUK using “ktrim = r k = 23 mink = 11 hdist = 1 minlength = 25 maxns = 3 tbo qtrim = r trimq = 1” (https://sourceforge.net/projects/bbmap/ (accessed on 1 March 2020)). High-quality reads were mapped on human genome (GRCh38) using hisat2 [8]. SAM files with mapped data were converted to BAM files and cleaned using SAMtools [9]. Statistical analysis and gene expression analysis were performed using R (3.5.1) (R Core Team (2013). R: A language and environment for statistical computing. R Foundation for Statistical Computing, Vienna, Austria. URL http://www.R-project.org/.), edger [10], ComplexHeatmap [11], and QoRTS packages. Enrichment analysis was performed using enrichR [12]. Data analysis source codes are available on Zenodo at https://zenodo.org/record/6920883 (accessed on 1 March 2020).

*Cell culture.* HCT116 cells were cultivated in McCoy’s 5A (1×) media (Gibco) containing 10% fetal bovine serum (Biochrom, Merck, Germany) and 50 µg/ml penicillin–streptomycin mix (Gibco, Thermo Fisher Scientific, Waltham, MA, USA) at 37 °C in humidified 5% CO_2_ conditions, exposed to normoxia (21% O_2_) or hypoxia (24 h, 1% O_2_) in an *Invivo200* hypoxic workstation (Ruskin Technologies).

*SR protein isolation from HCT116 cells.* SR proteins from HCT116 cells cultured under hypoxic (1% oxygen) or normoxic (21% oxygen) conditions were isolated as described earlier [13].

*Two-dimensional gel analysis.* A total of 30 µg of purified SR proteins prepared from normoxic or short-term (4 h) hypoxic HCT116 cells were subjected to two-dimensional analysis with IPGRunner strip (pH 3–10 L) using a ZOOM IPGRunner Mini-Cell (Invitrogen, Thermo Fisher Scientific, USA) apparatus according to the manufacturer’s instructions. Isoelectric focusing (200 V for 20 min, 350 V 10 min, 500 V 4 h, 2000 V for 2 h) was performed at 20 °C after a 12 h rehydration step. After the second-dimension separation on 12% polyacrylamide gel, proteins were transferred onto a nitrocellulose membrane, and blots were probed with anti-SR antibody (LifeSpan Biosciences, Seattle, WA, USA).

*HCT116 cell transfections*. Cells were seeded on 60 mm plates at a seeding density of 5.7 × 105 cells per plate and were allowed to recover for 24 h prior to transfection. Cells were transfected with PP1 (Ambion silencer select, ID s720), PP2A (Ambion silencer select, ID s10959), and control (Ambion, cat. 4390843, Thermo Fisher Scientific, Waltham, MA, USA) siRNA at a final concentration of 25 mM using RNAiMAX Transfection Reagent (Life Technologies, Thermo Fisher Scientific, Waltham, MA, USA) in appropriate culture medium, without antibiotics, containing 10% FBS according to the supplier’s protocol. After transfection, cells were allowed to recover for 18 h and subsequently exposed to normoxia (21% O_2_) or hypoxia (1% O_2_).

*RT-PCR.* Total RNA was isolated using the Quick-RNA™ MiniPrep kit (Zymo Research, USA) and cDNA was synthesized using the RevertAid H Minus First Strand cDNA Synthesis Kit (Thermo Fisher Scientific, USA). PCR was performed using the Mastercycler personal thermal cycler (Eppendorf, Hamburg, Germany). All PCR reactions were performed using DreamTaq DNA polymerase (Thermo Fisher Scientific) following the manufacturer’s recommendations. Used primers and their annealing locations are listed in Table 1. 18S RNA was used as a loading control. The PCR products were analyzed on 1.5% agarose gel in TBE buffer (90 mM Tris, 90 mM boric acid, 2 mM EDTA (pH = 8)), and band density was quantified using MultiGauge analysis software (Fuji). Results were expressed as a separate mRNA isoform relative ratio (%), with total mRNA isoform expression set as 100%. Statistical significance of all experiments was tested with a Mann–Whitney non-parametric U test using R statistical software based on a minimum of 3 independent experiments. Values of ** *p* < 0.05 and * *p* < 0.01 were considered statistically significant.

*Cell lysate.* Cell lysates were prepared using modified RIPA buffer (150 mm NaCl, 50 mm Tris-Cl pH 8.7, 1% Nonidet P-40, 0.5% sodium deoxycholate, 0.1% SDS, 2 mm dithiothreitol, 1 mm phenylmethylsulfonyl fluoride, and 0.2 mm *N*-ethylmaleimide) containing a protease inhibitor mixture (Roche Applied Science) and vortexed for 1 min, followed by centrifugation at 14,000 rpm for 30 min at 4 °C.

*Western blotting*. Whole cell lysates were separated on 12% SDS-PAAG, transferred on nitrocellulose membrane (GE Healthcare Life science), and blotted with appropriate primary and secondary antibodies.

Cell lysate was run on a 12% Tris-Glycine PAAG at 40 mA per gel (1.5 mm thickness) and then transferred onto nitrocellulose membranes (GE Healthcare). Membranes were blocked with 5% milk in TBST (20 mM Tris-HCl (pH 7.4), 150 mM NaCl, 0.05% Tween 20), and following two 15-m washing steps, were incubated with appropriate primary antibodies overnight at +4 °C. Membranes were then washed twice for 15 min with TBST and incubated with appropriate secondary antibodies for 2 h at room temperature. After two washing steps, membranes were visualized using TMB (3,3′,5,5′-tetramethylbenzidine) (Thermo Fisher Scientific). The antibodies used and their dilutions are listed in Table 2.

## 3. Results

*Cellular adaptation to hypoxia is not a fast process*. In our previous work, we showed that the hypoxic microenvironment regulates FAS exon 6 inclusion/exclusion to form mRNA. Under treatment of HCT116 cells for 24 h under hypoxic conditions, a shift in alternative FAS pre-mRNA splicing emerged, during which exon 6 was excluded from mRNA and the formation of anti-apoptotic sFAS mRNA was promoted [14].

**Figure 1:**
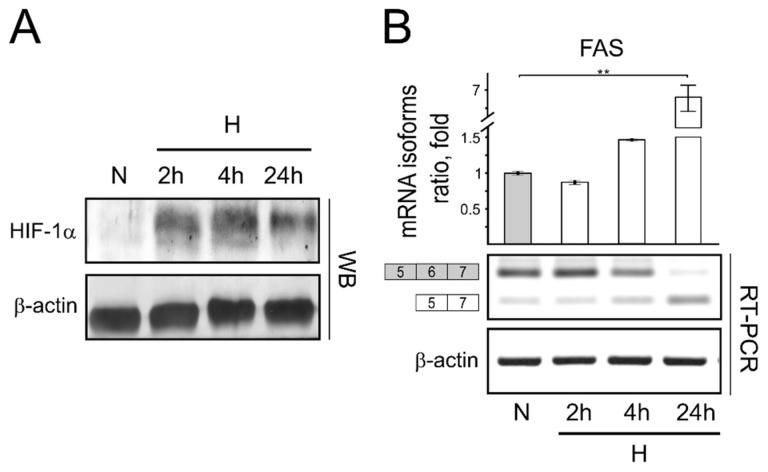
FAS exon 6 splicing pattern in short-term (2 h, 4 h) and long-term (24 h) hypoxia. (**A**) HIF-1 α protein cellular levels. (**B**) Reverse transcriptase PCR (RT-PCR) results of endogenous FAS/sFAS mRNA expression ratio in normoxic and hypoxic cells. Bar plot represents the quantification of FAS/sFAS isoform ratio of 3 independent experiments. The FAS/sFAS isoform expression ratio calculated in cells grown under normal oxygen conditions is taken as 1. Error bars represent the amount of spread between the extremes of the data, ** *p* < 0.05. N—cells cultivated under normal conditions; H—cells cultivated under hypoxic conditions for indicated time.

Since HIF-1α is the main regulator of hypoxic transcriptional response, we checked for HIF-1 protein levels in cells cultivated for 2 and 4 h under hypoxic conditions. Our investigation showed that HIF-1α protein levels can be detected in cells after just 2 h of cell cultivation in hypoxic conditions. After cell cultivation for 24 h in hypoxic conditions, the level of HIF-1 protein in cells slightly decreases (Figure 1A). However, our results revealed that even after 4 h under hypoxic conditions, splicing of apoptotic FAS mRNA isoform formation is still promoted (Figure 1B).

Such results show that in short-term hypoxia, the processes induced in cells are different compared to those observed in prolonged hypoxia. Based on these data, we decided to investigate how cells respond to short-term hypoxia in more detail.

**Figure 2:**
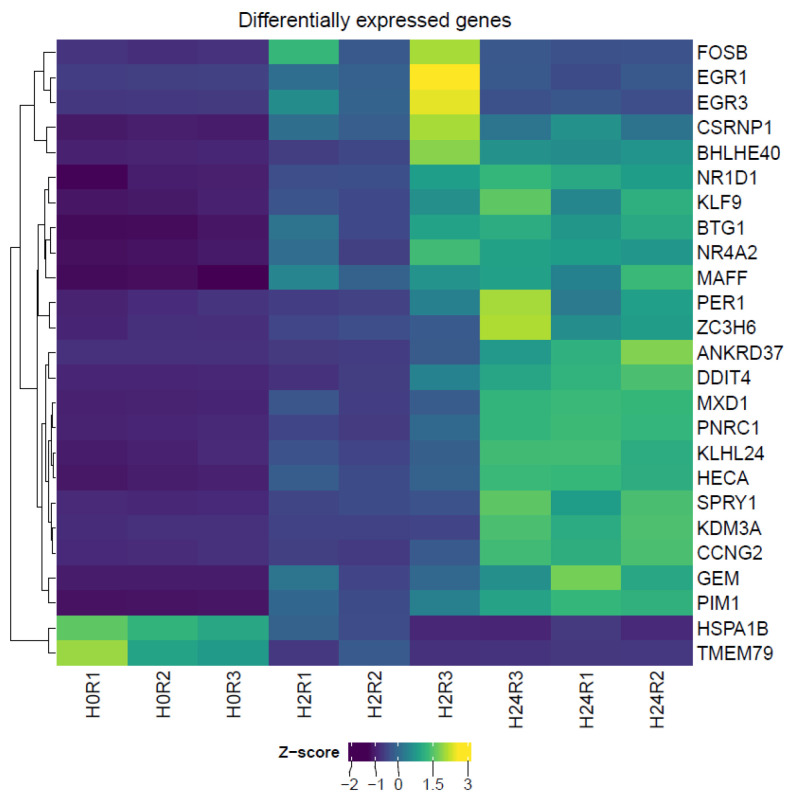
Z-score of normalized CPM values of differentially expressed genes. Genes with p-adjusted value < 0.05 and FC > 1.5 were selected and plotted using ComplexHeatmap package (Gu et al., 2016). H0—normoxic cells; H1, H2, and H24—cells incubated for 1 h, 2 h, and 24 h under hypoxic conditions, respectively.

*Computation analysis of RNA sequencing data.* We re-analyzed publicly available RNA-seq data of HCT116 cells treated with low oxygen (1%) levels and collected at different time points, i.e., 0 h, 1 h, 2 h, and 24 h (marked H0, H1, H2, and H24, respectively) (Memon et al., 2016 [7]). We used an unsupervised learning method, PCA (principal component analysis), to identify global data structure using gene expression data. PCA showed that samples collected after 24 h (H24) formed a separate cluster, with most of the variance explained by the first principal component. Early time point samples (H0, H1, and H2) clustered together and did not form individual clusters, indicating less variance between these time points, as expected. To investigate gene-level responses to hypoxia at early time points, we performed differential gene expression analysis using samples from H0 and H2 time points using the edge R package. As expected, we identified only a few differentially expressed genes using an FC threshold of 1.5 and an adjusted *p*-value (Benjamini–Hochberg correction) threshold of 0.05. Moreover, 23 out of the 25 genes showed upregulation in gene expression and only 2 showed downregulation (Figure 2). We further investigated the biological functions of the identified differentially expressed genes. We observed statistically significant enrichment of transcription regulation (GO:0006355, GO:0006357, GO:0045893, etc.), cellular processes (GO:1901216, GO:0045930), and developmental processes (GO:0048699, GO:0043124, GO:0045682), as well as DNA-binding molecular functions (GO:0003677, GO:0000977, GO:0000978, etc.) (Table S1).

**Figure 3:**
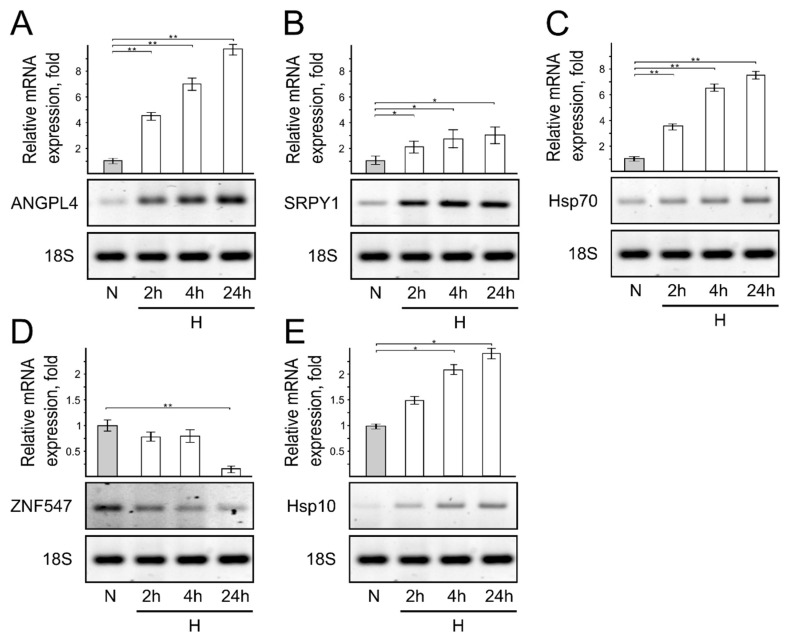
Short-term hypoxia influences ANGPL4, SRPY1, HSP70, ZNF54, and Hsp10 mRNA expression levels in normoxic and hypoxic cells. Reverse transcriptase PCR (RT-PCR) results of endogenous ANGPL4 (**A**), SRPY1 (**B**), Hsp70 (**C**), ZNF547 (**D**), and Hsp10 (**E**) gene mRNA expression in normoxic and hypoxic cells. Bar plot represents the quantification of mRNA expression of 3 independent experiments when mRNA expression calculated in cells grown under normal oxygen conditions is taken as 1. Error bars represent the amount of spread between the extremes of the data, ** *p* < 0.05, * *p* < 0.01. N—cells cultivated under normal conditions; H—cells cultivated under hypoxic conditions for indicated time (2, 4 h, and 24 h).

*Short-term hypoxia induces expression of genes associated with cell stress*. Computation analysis showed that short-term 2 h hypoxia induces the expression of genes associated with cell stress changes. Thus, to experimentally confirm the bioinformatics data analysis, we selected five genes, ANGPTL4 (angiopoietin-like 4) (Figure 3A), SRPY1 (sprout RTK signaling antagonist 1) (Figure 3B), ZNF547 (zinc finger protein 547) (Figure 3D), HSP70 (the 70 kilodalton heat shock protein) (Figure 3C), and HSP10 (the 10 kilodalton heat shock protein) (Figure 3E), whose changes were either high or statistically significant (Figure 3A–E), and investigated their expression in cells cultivated under hypoxic conditions for 2 h and 4 h. We used the 24 h time point in hypoxia as a positive control to show how gene expression changes under prolonged hypoxic conditions. The experimentally obtained results confirm the computation analysis data showing that even for cells cultivated for 2 h in hypoxia, the expression of analyzed genes is changed (Figure 3A–E).

Taken together, these results indicate that short-term hypoxia (2 h) induces the expression of genes known to be activated in case of oxidative and proteotoxic stress [15,16,17,18,19].

**Figure 4:**
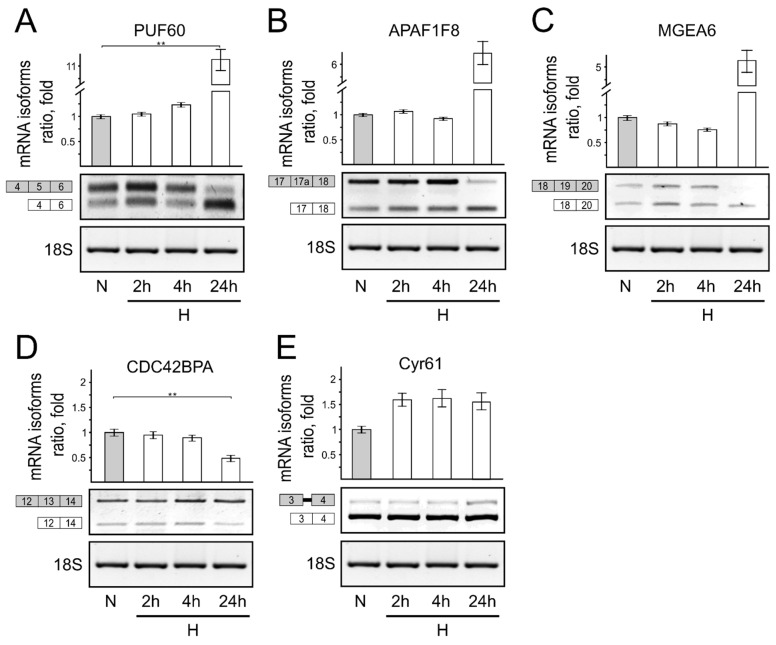
Influence of hypoxia on PUF60, APAF1F8, Cyr61, CDC42BPA, and MGEA6 gene alternative mRNA isoform formation. Reverse transcriptase PCR (RT-PCR) results of endogenous PUF60 (**A**), APAF1F8 (**B**), Cyr61 (**C**), CDC42BPA (**D**), and MGEA6 (**E**) genes and alternative mRNA isoform expression ratios in normoxic and hypoxic cells. Bar plot represents the quantification of alternatively spliced mRNA expression calculated in cells grown under normal oxygen conditions, taken as 1. Error bars represent the amount of spread between the extremes of the data, ** *p* < 0.05. N—cells cultivated under normal conditions; H—cells cultivated under hypoxic conditions for indicated time.

*Short-term hypoxia influences splicing of genes associated with cell stress and apoptosis*. Alternative pre-mRNA splicing is a vital process which helps cells adapt to changing environmental conditions [1,14,19]. Our bioinformatics analysis revealed the splicing of several genes in which normoxic and hypoxic cells were altered. Thus, we analyzed pre-mRNA alternative splicing patterns of genes from which translated proteins are involved in apoptosis (PUF60, APAF1F8) [20,21,22,23,24], cell stress (Cyr61) [25,26], cell migration (CDC42BPA) [27], and RNA processing (MGEA6) [28,29]. Our experimental data showed that short-term hypoxic conditions slightly influence alternative mRNA isoform formation patterns (Figure 4). It is established that after 24 h, cells are adapted to hypoxic conditions [30]. Thus, we used the 24 h time point as a control to show which mRNA isoforms are preferentially produced in cells after their adaptation to hypoxic conditions.

Our results clearly show that mRNA isoform formation from the same pre-mRNAs in cells under short-term hypoxic conditions and prolonged hypoxia are different (Figure 4A–E). It seems that short-term hypoxia in cells induces a process leading to apoptotic pathways, and only later, in order to survive under unfavorable conditions, is the hypoxia adaptation response induced.

*Short-term cellular hypoxia increases protein phosphatase but not protein kinase expression.* It is reported that hypoxic conditions increase the expression of protein kinases (CLK1-4, SRPK1, SRPK2) in cells [30,31,32]. Furthermore, growing evidence indicates that protein phosphatases (PP1, PP2A, PP2C) are important physiological regulators of cellular stress signaling [33].

**Figure 5:**
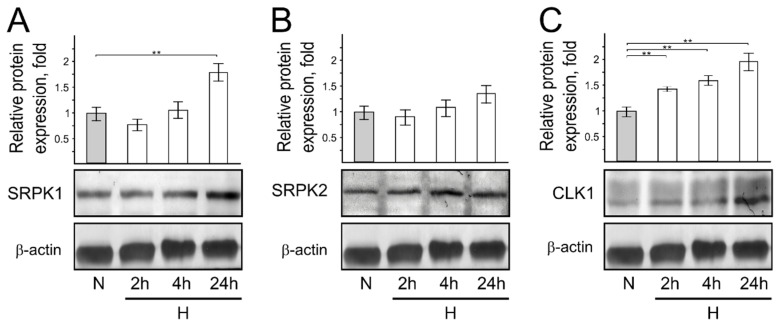
Protein kinase SRPK1, SRPK2, and CLK1 protein expression in normoxic and hypoxic HCT116 cells. Western blot showing SRPK1 (**A**), SRPK2 (**B**), and CLK1 (**C**) protein expression levels in normoxic and hypoxic cells. Bar plot represents the quantification of endogenous protein expression of 3 independent experiments when protein expression in cells grown under normal oxygen conditions is taken as 1. Error bars represent the amount of spread between the extremes of the data, ** *p* < 0.05. N—cells cultivated under normal conditions; H—cells cultivated under hypoxic conditions for indicated time (2 h, 4 h, and 24 h).

**Figure 6:**
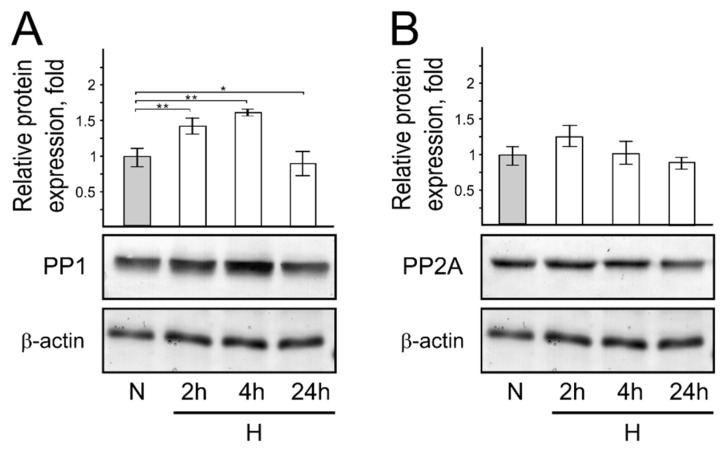
Protein phosphatase PP1 and PP2A protein expression in normoxic and hypoxic HCT116 cells. Western blot showing PP1 (**A**) and PP2A (**B**) protein expression levels in normoxic and hypoxic cells. Bar plot represents the quantification of endogenous protein expression of 3 independent experiments when protein expression in cells grown under normal oxygen conditions is taken as 1. Error bars represent the amount of spread between the extremes of the data, ** *p* < 0.05; * *p* < 0.01. N—cells cultivated under normal conditions; H—cells cultivated under hypoxic conditions for indicated time (2 h, 4 h, and 24 h).

Thus, we next investigated the expression of protein kinases and phosphatases in cells cultivated in short-term hypoxic conditions compared to cells cultivated in normal oxygen conditions. We found that short-term hypoxic conditions do not influence protein kinase expression levels, but the same protein expression is enhanced in cells cultivated under prolonged hypoxia (24 h) (Figure 5A–C). On the other hand, our results revealed an increase in the phosphatase expression level of cellular proteins grown under short-term hypoxia (Figure 6), as observed in stressed cells.

PP1 protein expression level, compared to protein expression in cells cultivated under normal environmental conditions, increased after 2 h and 4 h under hypoxic conditions (1.4- and 1.5-fold, respectively). After 24 h in hypoxia, the expression of PP1 was the same as observed in cells cultivated under normoxic conditions (Figure 6A). In cells cultivated for 2 h under hypoxic conditions, the expression of PP2A increased 1.2 times, while a slight reduction in the expression was observed after 24 h under hypoxic conditions (Figure 6B).

Such results may indicate that initially, in short-term hypoxic conditions, the same genes are expressed in cells that are activated during cell stress, and only later, in order to adapt to long-term hypoxic conditions, is the expression of genes necessary for cells to adapt to hypoxic conditions activated.

*Knockdown of expression of protein phosphatases influences the mRNA isoform formation ratio.* As we have discovered that short-term hypoxia induces PP1 and PP2A protein expression levels, we have raised the question if these protein phosphatases could be critical in short-term hypoxia-initiated changes in mRNA formation patterns. To answer this question, we investigated whether the reduction in PP1 and PP2A cellular expression would have any influence on mRNA isoform formation patterns in HCT116 cells cultivated in hypoxia.

**Figure 7:**
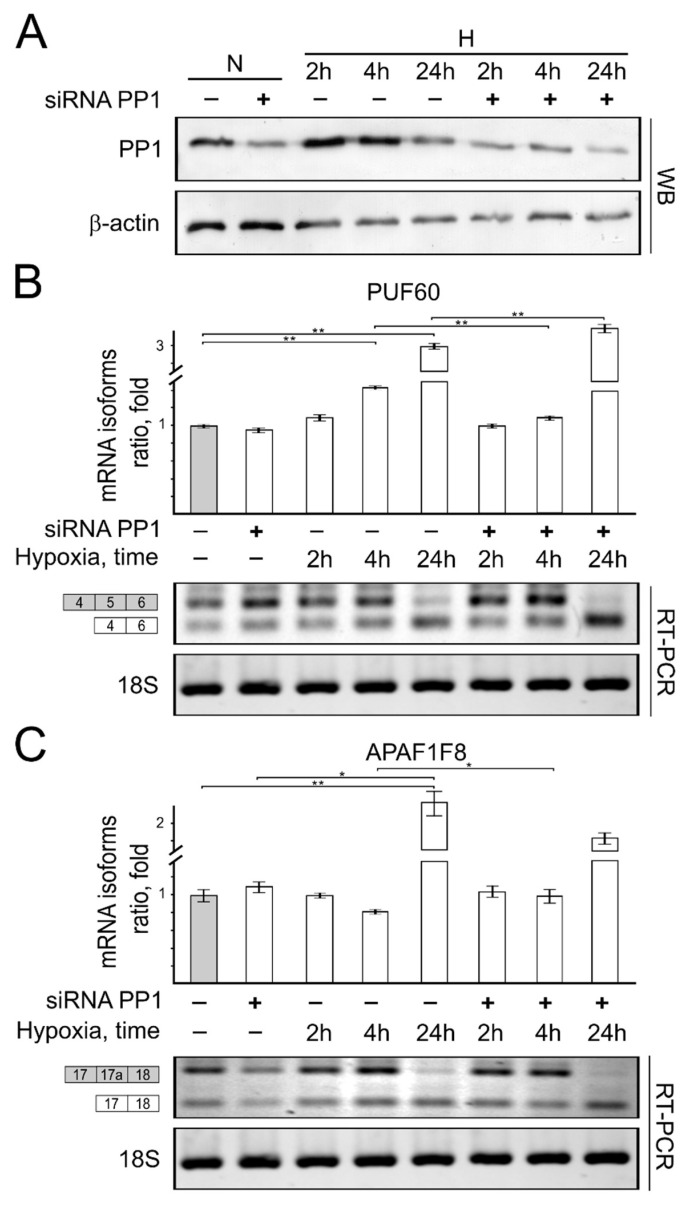
The role of PP1 protein expression for PUF60 and APAF1F8 splicing in normoxic and hypoxic HCT116 cells. Western blot showing PP1 (**A**) protein expression levels in normoxic and hypoxic cells. Reverse transcription PCR (RT-PCR) results showing PUF60 and APAF1F8 gene alternative mRNA isoform expression ratio in reduced PP1 (**B**,**C**) protein levels expressing normoxic and hypoxic HCT116 cells. Bar plot represents the quantification of endogenous protein expression of 3 independent experiments when protein expression in cells grown under normal oxygen conditions is taken as 1. Error bars represent the amount of spread between the extremes of the data, ** *p* < 0.05; * *p* < 0.01. N—cells cultivated under normal conditions; H—cells cultivated under hypoxic conditions for indicated time (2 h, 4 h, and 24 h).

**Figure 8:**
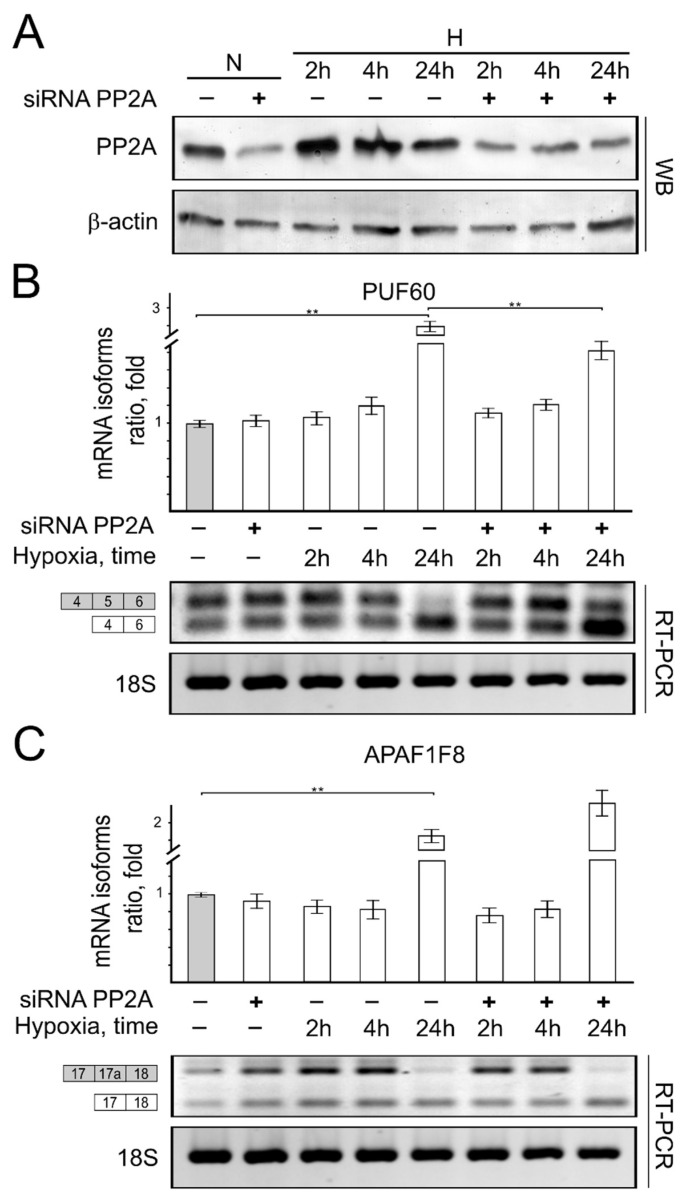
The role of PP2A protein expression for PUF60 and APAF1F8 splicing in normoxic and hypoxic HCT116 cells. Western blot showing PP2A (**A**) protein expression levels in normoxic and hypoxic cells. Reverse transcription PCR (RT-PCR) results showing PUF60 and APAF1F8 genes and alternative mRNA isoform expression ratios in reduced PP2A (**B**,**C**) protein levels expressing normoxic and hypoxic HCT116 cells. Bar plot represents the quantification of endogenous protein expression of 3 independent experiments when protein expression in cells grown under normal oxygen conditions is taken as 1. Error bars represent the amount of spread between the extremes of the data, ** *p* < 0.05. N—cells cultivated under normal conditions; H—cells cultivated under hypoxic conditions for indicated time (2 h, 4 h, and 24 h).

Our results show that the decrease in PP1 (Figure 7) and PP2 (Figure 8) protein cellular levels had only a slight effect on PUF60 (Figure 7B and Figure 8B) and APAF1F8 (Figure 7B and Figure 8B) alternatively spliced mRNA isoform formation patterns in cells cultivated under normoxic conditions.

mRNA isoform formation analysis of the PUF60 gene showed that in transient hypoxic conditions, the decrease in PP1 protein levels lowers the PUF60 mRNA alternatively spliced isoform ratio up to 0.4-fold (40%) (Figure 7B). The opposite results were obtained when cells were incubated under hypoxic conditions for 24 h, when the decrease in PP1 protein levels increased the alternative mRNA isoform formation ratio of the PUF gene by 1.2-fold (Figure 7B).

Lower PP1 protein levels (Figure 7) in short-term hypoxia (2 h, 4 h) increased the formation ratio of alternatively spliced APAF1F8 mRNA isoforms by (Figure 7C) 1.2-fold. On the other hand, in prolonged hypoxic conditions, a 1.5-fold increase in the APAF1F8 mRNA alternatively spliced isoform formation ratio was detected (Figure 7C).

Knockdown of PP2A protein cellular levels (Figure 8) had little influence on tested gene splicing in short-term hypoxia-treated cells. No effects of PP2A protein expression changes on alternative mRNA isoform formation were observed for PUF60 and APAF1F8 genes in cells cultivated under short-term hypoxic conditions (Figure 8). Under prolonged hypoxic conditions, a 1.5-fold decrease in PUF60 and a 1.2-fold increase in APAF1F8 in mRNA alternative isoform expression ratios were detected (Figure 8B,C).

Such results indicate that initially, under short-term hypoxia, an increase in protein phosphatase cellular expression is important for changing mRNA isoform formation. In particular, an increase in protein phosphatase cellular levels is important in the early stages of hypoxia. Moreover, the results highlight that not only protein kinase expression levels in cells are important for cellular adaptation to hypoxic conditions, but protein phosphatase cellular levels also play an important role.

**Figure 9:**
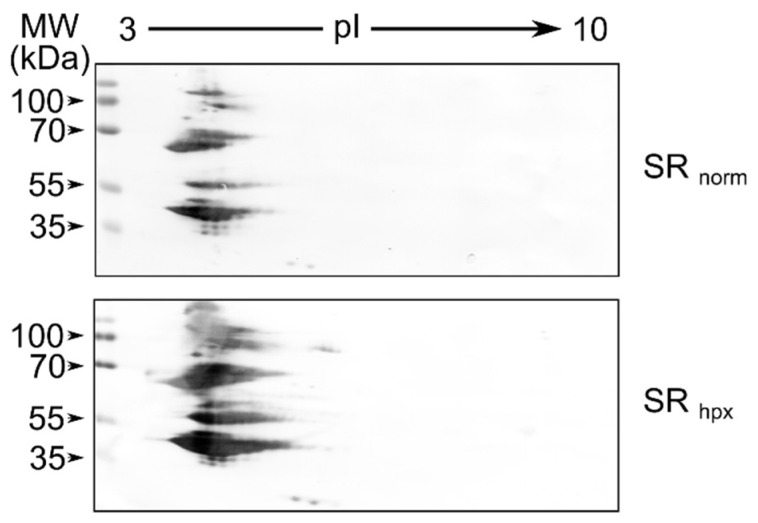
Hypoxia induces modifications of SR proteins. Western blot of two-dimensional gel electrophoresis of SR proteins isolated from HCT116 cells cultivated under normoxic (SR norm) and hypoxic (SR hpx) conditions for 4 h.

*Enhanced protein phosphatase levels influence the protein modification level.* It has been known for a long time that protein phosphatases such as PP1 and PP2a influence the phosphorylation of splicing factors, such as SR protein C-terminal arginine–serine-rich (RS) domain level [34]. In order to determine if the enhancement of protein phosphatase expression in short-term hypoxia influences SR protein modification levels, we isolated SR proteins from cells cultured under normoxic conditions or for 4 h under hypoxic conditions and ran a 2D gel. The 2D gel Western blot, using anti-SR antibody, showed that some fraction of SR proteins are shifted to the basic side, indicating that the enhancement of phosphatase activity in cells influences the modification of proteins involved in pre-mRNA splicing (Figure 9).

The obtained results indicate that the enhanced levels of protein phosphatases in cells after exposing them to short-term hypoxic conditions influence protein modification levels. Moreover, such results could indicate that the expression of protein kinases, as well as protein phosphatases, is needed for cells to adapt to prolonged hypoxic conditions.

## 4. Discussion

Cells are constantly suffering endogenous and exogenous stresses that affect the integrity of the genetic material [35]. Many environmental variations impose stresses that can damage or kill the organism in the absence of an appropriate response [36]. However, little is known about the signals that emanate from stressed cells to enable a coordinated adaptive response across tissues, organs, and the whole organism [36].

The expressions of many genes that are important for basal metabolism and core cellular components, such as ribosomal proteins, splicing factors, and cytoskeleton proteins, were changed in different cell stress conditions [36]. In the current work, we show that short-term hypoxia induces the expression of genes known to be activated in response to oxidative and proteotoxic stress [15,16,17,18,19]. Our data indicate that to deal with inadequate oxygenation, cells initially employ adaptive measures and change the expression of the same genes observed to be altered in stressed cells.

It is known that prolonged hypoxia changes multiple cellular processes. Available data show that in prolonged cellular hypoxia, the expression of protein kinases is enhanced and that this enhancement is driven by transcription factor HIF-1 [30,31,32]. Despite the fact that in short-term hypoxic cells, HIF-1α protein is stabilized, the expression of protein phosphatases, not protein kinases, is increased in short-term hypoxic cells.

Our experimental data show that alternative mRNA isoform formation patterns from the same pre-mRNAs in cells under short-term hypoxic conditions and prolonged hypoxia differ. This could be explained by short-term and prolonged hypoxia initiating different cell response mechanisms.

Available scientific data show that under non-stressed conditions, the phosphatase and counteracting kinase activities are balanced [33]. It is reported that protein phosphatases are important physiological regulators of cellular stress signaling [6,33]. For example, it is shown that oxidative stress may induce PP2A-dependent dephosphorylation of the proteins. It is also known that PP2A inhibitors may block oxidative stress-mediated ERK5 activation [37,38]. In our studies, we found that short-term hypoxia, like cell stress, induces the expression of protein phosphatases, which are known as important physiological regulators of cellular stress signaling [6,33]. Despite the fact that the modification level of splicing factors changes in short-term hypoxic cells, our studies did not reveal a significant influence of these changes on pre-mRNA splicing. However, in prolonged hypoxic conditions, alternative splicing pattern alterations are observed. Such late processes of cellular adaptation to prolonged hypoxic conditions can be explained by the fact that a longer time is needed to reorganize the cellular processes.

Our results also show that the enhanced expression of protein kinases and protein phosphatases is needed for cells in order to adapt to prolonged hypoxic conditions.

In general, the results obtained in this study show that in response to short-term and prolonged hypoxic conditions, different response mechanisms are induced in cells.

Cellular hypoxia is associated with multiple diseases, such as solid tumors. It is known that solid tumors possess hypoxic regions. It seems that in solid tumors, there are not only hypoxic regions but also regions in which cellular mechanisms operate similarly to cell stress response mechanisms. Thus, our study results also give some explanation as to why drugs used in cancer treatment sometimes have a weak effect.

Of course, to better understand the influence of hypoxia on cellular processes, more studies should be carried out, but this is the first attempt to better understand the influence of transient hypoxia on cellular processes.

## 5. Limitations of the Study

This is the first attempt to show that short-term hypoxic conditions in cells induce processes that are observed in stressed cells. It seems that initially, under hypoxic conditions, the cell stress response mechanism is induced, and only later do cells induce a mechanism enabling adaptation to hypoxic conditions. More studies should be conducted in order to understand short-term hypoxia-induced cellular processes, and if they are the same as those observed in stressed cells.

## Figures and Tables

**Table 1:** PCR primers.

Primer Name	Primer Sequence	Product Length
18S	*Fw* AACTCACTGAAGATGAGGTG *Rev* CAGACAAGGCCTACAGACTT	305 bp
actin	*Fw* GACAGGATGCAGAAGGAGAT *Rev* TTGCTGATCCACATCTGCTG	146 bp
ANGPL4	*Fw* GATGGCTCAGTGGACTTCAACC *Rev* TGCTATGCACCTTCTCCAGACC	100 bp
SRPY1	*Fw* GAAAGAGGACCTGACACAGCAC *Rev* CTCTCAGCAGAGCAAAGGCACT	162 bp
ZNF547	*Fw* AGGCTCAGAGATTGCTGTACCG *Rev* CACTCCTACAGAAACACCTGGC	125 bp
Hsp70	*Fw* ACCTTCGACGTGTCCATCCTGA *Rev* TCCTCCACGAAGTGGTTCACCA	122 bp
Hsp10	*Fw* CTCCCAGAATATGGAGGCACC *Rev* TGGAATGGGCAGCATCATGT	139 bp
FAS	*Fw* GTGAACACTGTGACCCTTGC *Rev* CCTTGGTTTTCCTTTCTGTGC	205 bp 142 bp
PUF60	*Fw* GCCAAGAAGTACGCCATGG *Rev* GTAGACGCGGCACATGATG	189 bp 138 bp
APAF1F8	*Fw* GTGAAGTGTTGTTCGTGGTCTG *Rev* CATCACACCATGAACCCAAC	322 bp 193 bp
Cyr61	*Fw* GGCAGACCCTGTGAATATAA *Rev* CAGGGTTGTCATTGGTAACT	612 bp 481 bp
MGEA	*Fw* CTGAAACAGAGCTTAAATTTGAAC *Rev* CTGGCGGAGGAAACATCATCC	366 bp 237 bp
CDC42BPA	*Fw* GCTAATGCTGTGAGGCAAGAAC *Rev* GCTCACTCTGTTCACGTAGCTT	415 bp 172 bp

**Table 2:** Antibodies used.

Antibody Name	Manufacturer, Cat. Number	Dilution Used
anti-HIF-1α, mouse mAb	BD transduction laboratories, 610959	1:500
anti-SRPK1, rabbit mAb	Abcam, ab131160	1:1000
anti-SRPK2, rabbit pAb	Abcam, ab67993	1:1000
anti-CLK1, rabbit pAb	Abcam, ab74044	1:300
anti-PP1, rabbit mAb	Abcam, ab134947	1:1000
anti-PP2A, rabbit mAb	Abcam, ab32104	1:1000
anti-SR, mouse mAb	Lifespan Biosciences, LS-C75961	1:1000
anti-β actin, mouse mAb	Abcam, ab3280	1:5000
Goat anti-mouse, HPR conjugated	Dako, P 044701	1:2000
Goat anti-rabbit, HPR conjugated	Dako, P 0448801	1:2000

## Data Availability

Not applicable.

## Supplementary Materials

The following supporting information can be downloaded at: https://www.mdpi.com/article/10.3390/genes13091596/s1, Table S1: GO enrichment.
